# In through the Out Door: A Functional Virulence Factor Secretion System Is Necessary for Phage Infection in Ralstonia solanacearum

**DOI:** 10.1128/mbio.01475-22

**Published:** 2022-10-31

**Authors:** André da Silva Xavier, Alessandra G. de Melo, Connor G. Hendrich, Denise M. Tremblay, Geneviève M. Rousseau, Pier-Luc Plante, Katrina T. Forest, Poliane Alfenas-Zerbini, Caitilyn Allen, Sylvain Moineau

**Affiliations:** a Departamento de Fitopatologia, Instituto de Biotecnologia Aplicada à Agropecuária (BIOAGRO), Universidade Federal de Viçosa, Viçosa, MG, Brazil; b Département de biochimie, de microbiologie, et de bio-informatique, Faculté des sciences et de génie, Université Laval, Québec, Quebec, Canada; c Groupe de Recherche en Écologie Buccale, Faculté de médecine dentaire, Université Laval, Québec, Quebec, Canada; d Department of Plant Pathology, University of Wisconsin—Madison, Madison, Wisconsin, USA; e Félix d'Hérelle Reference Center for Bacterial Viruses, Université Laval, Québec, Quebec, Canada; f Département de médecine moléculaire, Faculté de médecine, Université Laval, Québec, Quebec, Canada; g Department of Bacteriology, University of Wisconsin—Madison, Madison, Wisconsin, USA; h Departamento de Microbiologia, Instituto de Biotecnologia Aplicada à Agropecuária (BIOAGRO), Universidade Federal de Viçosa, Viçosa, MG, Brazil; i National Research Institute on Plant-Pest Interactions, Universidade Federal de Viçosa, Viçosa, MG, Brazil; University of Nebraska—Lincoln

**Keywords:** fitness cost, phage resistance, *Ralstonia solanacearum*, bacterial wilt, virus-host interactions, pseudopilin, general secretion pathway

## Abstract

Bacteriophages put intense selective pressure on microbes, which must evolve diverse resistance mechanisms to survive continuous phage attacks. We used a library of spontaneous Bacteriophage Insensitive Mutants (BIMs) to learn how the plant pathogen Ralstonia solanacearum resists the virulent lytic podophage phiAP1. Phenotypic and genetic characterization of many BIMs suggested that the R. solanacearum Type II Secretion System (T2SS) plays a key role in phiAP1 infection. Using precision engineered mutations that permit T2SS assembly but either inactivate the T2SS GspE ATPase or sterically block the secretion portal, we demonstrated that phiAP1 needs a functional T2SS to infect R. solanacearum. This distinction between the static presence of T2SS components, which is necessary but not sufficient for phage sensitivity, and the energized and functional T2SS, which is sufficient, implies that binding interactions alone cannot explain the role of the T2SS in phiAP1 infection. Rather, our results imply that some aspect of the resetting of the T2SS, such as disassembly of the pseudopilus, is required. Because R. solanacearum secretes multiple virulence factors via the T2SS, acquiring resistance to phiAP1 also dramatically reduced R. solanacearum virulence on tomato plants. This acute fitness trade-off suggests this group of phages may be a sustainable control strategy for an important crop disease.

## INTRODUCTION

In the ongoing arms race between phages and bacteria, virus-mediated selection plays a central role in maintaining bacterial diversity (1, 2). This selective pressure drives rapid molecular evolution as part of the natural cycles of adaptation and counter adaptation (3 to 5). The prevalence of a genotype is determined by the relative adaptive advantages and biological cost of its cognate functions (6). Bacterial hosts resist viruses directly with mechanisms like the broadly distributed CRISPR-Cas systems (7) restriction-modification (R-M) (8), and abortive infection (Abi) systems (9). Additionally, viral resistance phenotypes can result from host mutations that alter factors essential for primary interactions with the phage (10), such as viral adsorption (11, 12).

The Gram-negative betaproteobacterium Ralstonia solanacearum (R. solanacearum) is a widely distributed plant pathogen that belongs to a bacterial complex called Ralstonia solanacearum species complex (RSSC), consisting of three species, Ralstonia syzygii, R. solanacearum, and Ralstonia pseudosolanacearum (13). Originally, the RSSC isolates were subdivided into four genotypically different phylotypes and classified according to their geographical origins. Currently, the isolates belonging to phylotypes I and III are classified as *R. pseudosolanacearum*, and the isolates from the phylotypes II and IV as R. solanacearum and *R. syzygii*, respectively (13, 14). R. solanacearum strains form a heterogeneous group that causes lethal bacterial wilt disease in more than 200 plant species, including economically important crops like potato, banana, tobacco, and tomato (14 to 18).

Efforts to control bacterial wilt with bacteriophages have had mixed results because R. solanacearum populations are often phage resistant, apparently using multiple strategies to escape viral infection (19 to 21). Some R. solanacearum isolates carry canonical CRISPR-Cas systems, but these are apparently ineffective in their native state (22). The occurrence of phage-insensitive strains suggests R. solanacearum has other viral evasion strategies. To better understand the natural anti-phage strategies in R. solanacearum, we generated a library of spontaneous bacteriophage insensitive mutants (BIMs) by exposing wild-type (WT) R. solanacearum strain CFBP2957 (Phylotype 2A) to the virulent podophage phiAP1. Two of these BIMs, BIM4 and BIM30, were previously characterized (22) and shown to allow phage adsorption but not phage DNA replication.

**FIG 1:**
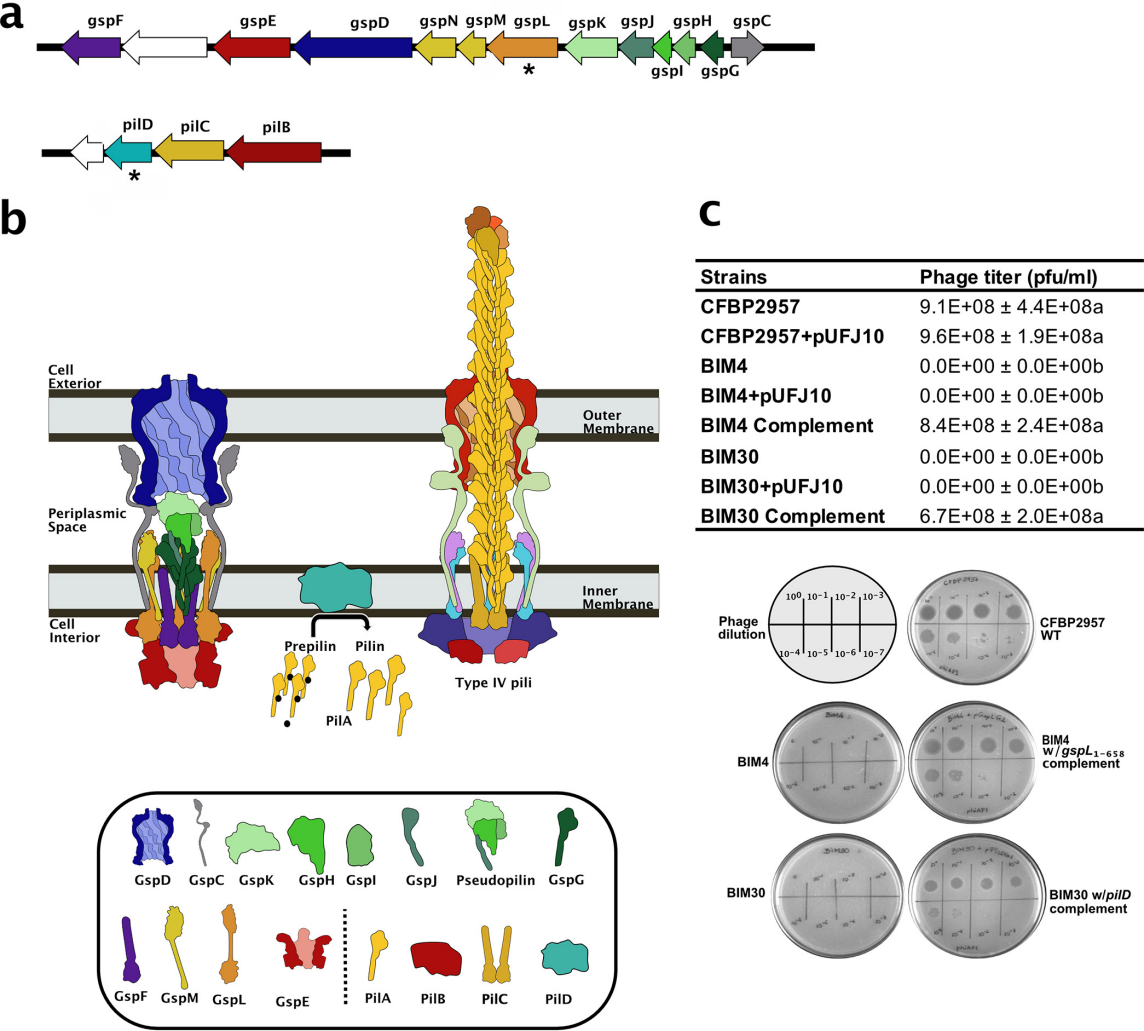
Mutations in *gspL* and *pilD* confer resistance to phage phiAP1. (a) Genomic location of the unique mutations in R. solanacearum BIM4 and BIM30. BIM4 contains a mutation in *gspL*, a gene coding for an inner membrane protein of the T2SS. BIM30 contains a mutation in *pilD* coding for a prepilin peptidase of the T4P. Asterisk indicates mutated genes in the BIMs. (b) Structure of the T2SS. The inner membrane complex is composed of the cytoplasmic ATPase GspE (shown in red) and the inner membrane proteins GspC, GspF, GspL, and GspM. GspN is not pictured. GspC (shown in gray) links the inner membrane complex to the outer membrane complex, which is composed of the outer membrane secretin GspD (shown in blue). GspE uses the energy from ATP hydrolysis to assemble the pseudopilus, which is a polymer of the major pseudopilin GspG (shown in dark green) capped with the minor pseudopilins GspH, GspI, GspJ, and GspK (shown in shades of light green). The growth of the pseudopilus pushes T2SS cargo out of the cell. The T2SS is structurally related to the type IV pilus, shown on the right. PilA (shown in yellow) processed by PilD are added to the pilus. (c) The table shows the titration results of phage phiAP1 on CFBP2957, BIM4, and BIM30, in addition to the strains with empty pUFJ10 and the BIMs complements. The values are the average of two double-layer plaque assay independent experiments performed in triplicates ± standard deviation. Different letters in the same column are significantly different at *P* < 0.05 (ANOVA). Both BIM4 and BIM30 are resistant to phage phiAP1 infection, and complementing the mutants with the first 658 nucleotides (219 amino acids) of *gspL* or full-length *pilD*, respectively, restores their phage sensitivity. Ten-fold dilutions of a phage phiAP1 lysate were spotted onto a lawn of each bacterial strain in the spot test.

Here, we identify the genetic determinants of phage resistance in several BIMs derived from R. solanacearum CFBP2957. One system implicated was the Type II Secretion System (T2SS). The T2SS is a complex, highly dynamic molecular machine that transports folded proteins from the periplasm to the exterior of the bacterial cell (Fig. 1). This system is composed of an outer membrane protein complex, the secretin, which is made of 15 GspD subunits; an inner membrane platform; and transenvelope connecting proteins. The inner membrane platform (23, 24) is formed by the transmembrane proteins GspL, GspM, and GspF, as well as the cytoplasmic ATPase GspE. ATP hydrolysis by GspE provides the energy that drives assembly of the pseudopilus. This pseudopilus is a polymer of the major pseudopilin GspG with a terminal cap composed of four minor pseudopilins (23, 25). Growth of the pseudopilus guides the protein cargo into the periplasmic vestibule of the outer membrane secretin complex, and from there it leaves the cell via the secretin pore domain. Prior to their addition to the growing pseudopilus, charged leader peptides must be removed from prepseudopilins by a prepilin peptidase (26). Interestingly, we discovered that phage phiAP1 needs a functional T2SS, which R. solanacearum uses to secrete virulence factors. We found that function-destroying mutation of T2SS components provided phage resistance but imposed a severe fitness cost because R. solanacearum BIMs had significantly reduced virulence on tomato plants. Furthermore, we showed that the PilD prepilin peptidase plays an active role in both type IV pili (T4P) and in the T2SS of R. solanacearum.

## RESULTS

### Mutations in the T2SS and T4P in BIMs of R. solanacearum.

To understand the mechanism of phage resistance in R. solanacearum mutants, we first sequenced the genomes of two BIMs (BIM4 and BM30) that are fully resistant to phage phiAP1 as well as of the phage-sensitive WT parent strain CFBP2957. Several single nucleotide mutations and deletions were identified in the two BIMs compared to the WT. To map the mutations involved in phage resistance, we focused on the few nonsynonymous mutations (Table S1). BIM4 contained a 3-bp deletion that removed a leucine at position 65 in the membrane protein GspL (481 amino acids), which is involved of the general secretion pathway. Specifically, GspL is a part of the T2SS inner membrane complex and participates in the formation of the pseudopilus (Fig. 1a and b) (23). In BIM30, a 4-bp deletion caused a frameshift after the 42nd of the 290 amino acids of the prepilin peptidase (PilD). PilD processes prepilins before they are added to the type IV pilus (T4P), a structure involved in R. solanacearum twitching motility and virulence.

10.1128/mbio.01475-22.1 TABLE S1 Mutation in the BIMs of Ralstonia solanacearum strain CFBP2957. Download Table S1, DOCX file, 0.1 MB. Copyright © 2022 Xavier et al. 2022 Xavier et al. https://creativecommons.org/licenses/by/4.0/ This content is distributed under the terms of the Creative Commons Attribution 4.0 International license.

### Phage sensitivity is restored by complementing the BIM mutations.

To confirm that the mutations in *gspL* and *pilD* were responsible for the phage resistance phenotype, we first separately cloned the WT *gspL* and *pilD* genes from R. solanacearum CFBP2957 into the low-copy replicative plasmid pUFJ10 (27) to generate plasmids pGspL and pPilD. The expression of each WT gene is under the control of the kanamycin resistance cassette promoter in pUFJ10. While cloning *pilD* was straightforward and expected sequenced-confirmed clones were readily obtained, we could not clone the complete *gspL* despite multiple attempts. We did, however, succeed in cloning a truncated version of *gspL*, which contained the first 219 codons (the mutation in BIM4 is in codon 65). These cloning difficulties suggested that this gene, coding for a membrane protein, is detrimental for E. coli growth. Still, plasmids pPilD and pGspL_219_ (containing a truncated version) were both successfully transformed into R. solanacearum BIM30 and BIM4, respectively. Of significant interest, both complemented strains (BIM4::pGspL_219_ and BIM30::pPilD) were sensitive to phage phiAP1, restoring the phage-sensitive phenotype of the WT strain CFBP2957 (Fig. 1c). In a separate experiment, *pilD* was also cloned under its native promoter into pUFJ10, and this construct also restored sensitivity to phage phiAP1. These results confirmed that R. solanacearum PilD and GspL are essential for phage phiAP1 to complete its infection cycle.

### BIM4 and BIM30 are defective in Type II secretion, twitching motility, or both.

**FIG 2:**
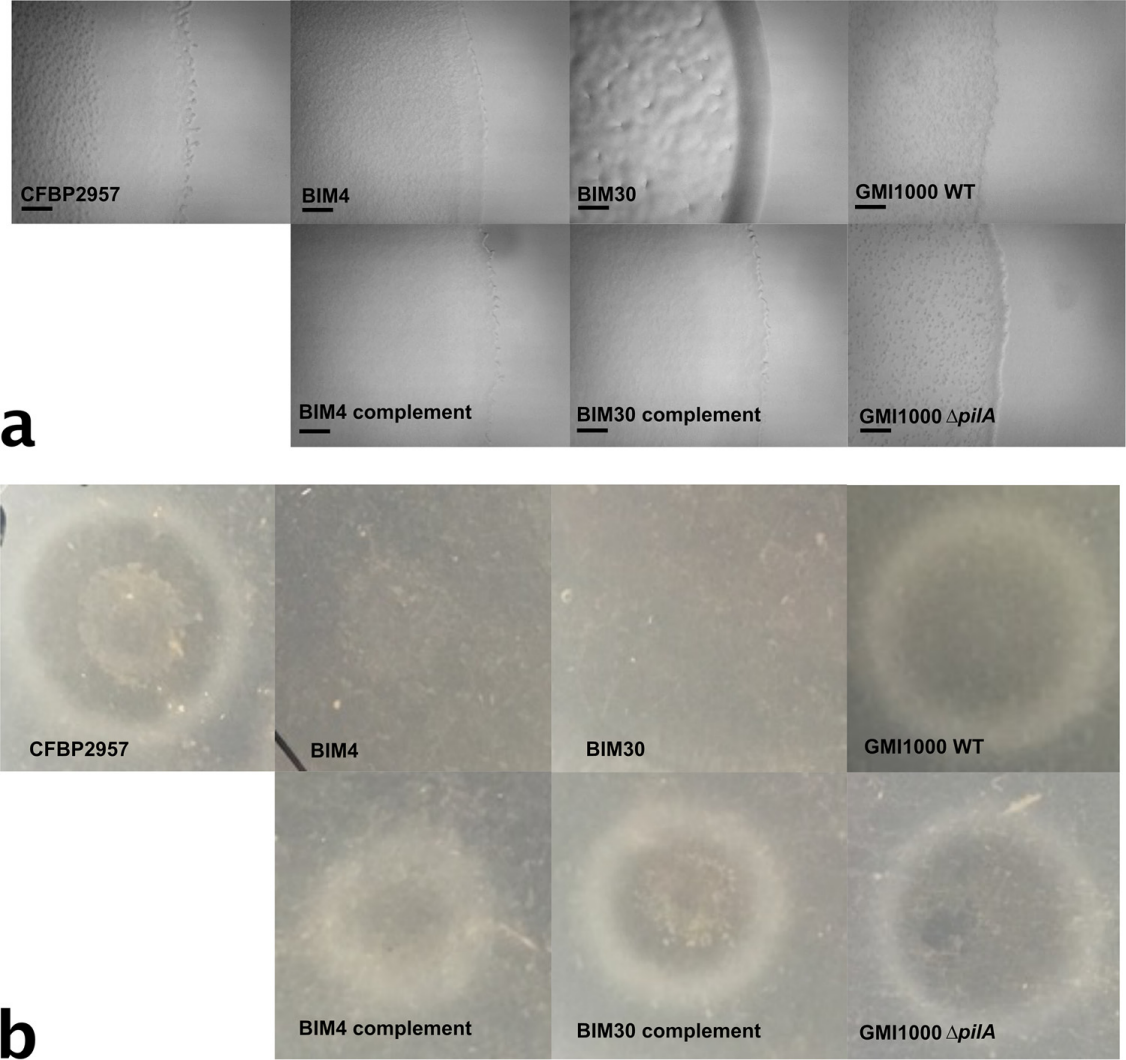
R. solanacearum BIM4 and BIM30 are both deficient in T2SS, but only BIM30 is deficient in T4P-mediated twitching motility. (a) Colony margins of R. solanacearum CFBP2957 WT, BIM4, and BIM30 grown on low-percentage (0.08%) agar plates. GMI1000 and a twitching deficient mutant (GMI1000 *ΔpilA*) are included as controls. The colony margins of WT and BIM4 at low cell densities were diffuse and undefined. In contrast, microcolonies of BIM30 and *ΔpilA* coalesced earlier, forming compact, defined masses even at low densities. BIM30 was deficient in twitching motility, but BIM4 was not. Scale bars represent 0.1 mm. (b) Secretion of the T2SS polygalacturonase enzymes by WT R. solanacearum CFBP2957, BIM4, BIM30, WT GMI1000, and GMI1000 *ΔpilA*. Strains were plated on minimal media plates containing polygalacturonic acid. After 24 h of growth, the colonies were rinsed away, and the plates were flooded with 2N HCl to reveal zones of clearing made by the polygalacturonase activity. Both BIM4 and BIM30 were unable to export polygalacturonase enzyme, indicating they have defects in the T2SS.

Because *gspL* and *pilD* are predicted to be involved in the activity of T2SS and T4P, respectively, we assayed these two functions in BIM4 and BIM30. Twitching motility was used to assess T4P functionality by spotting each strain on low-percentage agar plates and observing colony margins after 16 h of growth (28). The edges of the BIM4 and WT colonies had the diffuse and reticulate margins typical of R. solanacearum twitching motility (Fig. 2a) (28). In contrast, the BIM30 colony rapidly developed thick, defined edges, similarly to a twitching-defective control strain lacking the major pilin PilA (Fig. 2a). These results indicate that the loss of *pilD* in BIM30 impairs R. solanacearum twitching motility.

As an indicator of T2SS function, we tested BIM4 and BIM30 for activity of the type II secreted enzyme polygalacturonase. When grown on agar plates containing polygalacturonic acid substrate, the T2SS-positive WT R. solanacearum strain CFBP2957 produced a clearing zone due to the secretion of this enzyme (29). Neither BIM4 nor BIM30 produced a clearing zone on polygalacturonic acid plates, indicating a nonfunctional T2SS (Fig. 2b). In Pseudomonas aeruginosa, PilD processes not only pilins for the T4P, but also plays a role in the T2SS (30). The loss of these activities in BIM30 strongly suggests that PilD plays the same role in R. solanacearum T2SS as it does in P. aeruginosa.

### Phage phiAP1 does not directly depend on T4P for R. solanacearum infection.

**FIG 3:**
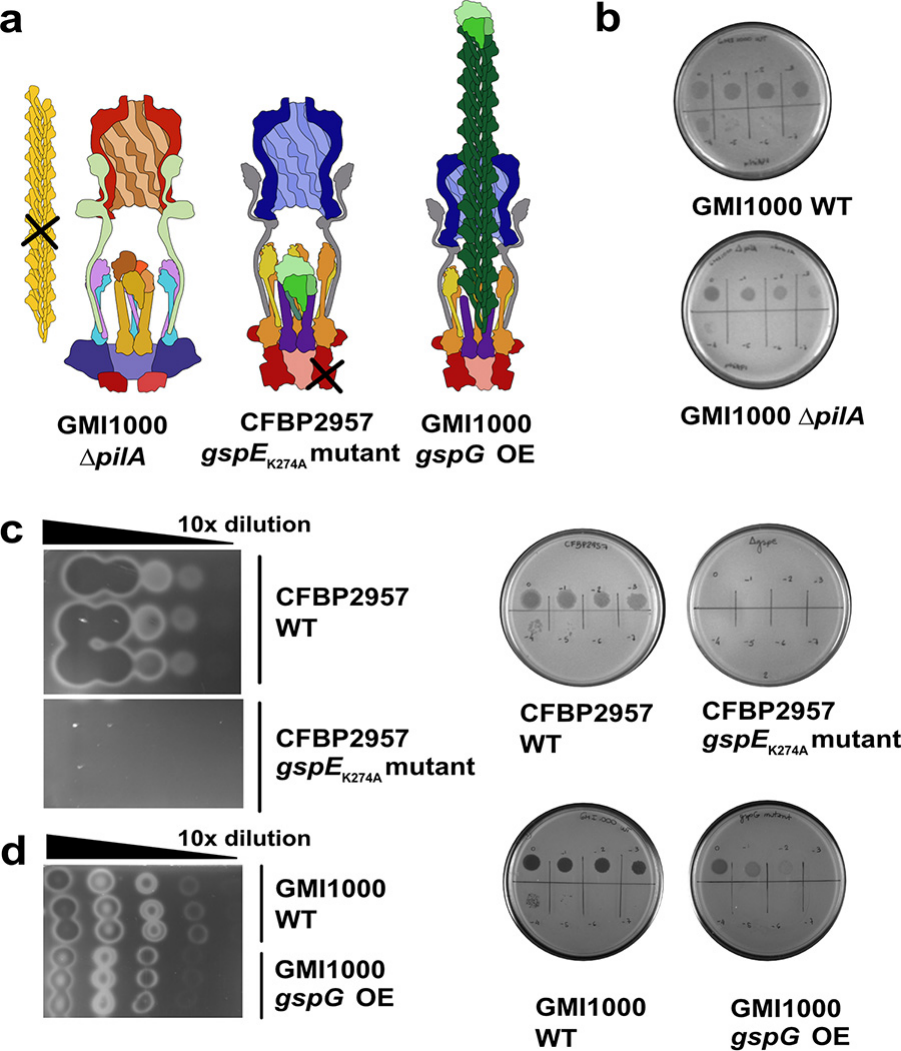
Phage phiAP1 infection depends on an active T2SS, but not the T4P. (a) Predicted effects of two T2SS deficient mutants. (b) Serial dilutions (10-fold) of phage phiAP1 lysate were spotted onto a lawn of either GMI1000 WT or GMI1000 *ΔpilA*. The *ΔpilA* mutant was as sensitive to phage phiAP1 as the WT, indicating that T4P is not required for phiAP1 infecion. The R. solanacearum *gspE_K274A_* mutant contains a single mutation in the ATP binding domain of the inner membrane ATPase GspE, shown in red. When GspE is unable to bind and hydrolyze ATP, it cannot add pseudopilins to the pseudopilus, and the system cannot export any cargo. The *R. pseudosolanacearum* GMI1000 *gspG* OE mutant contains an additional copy of the major pseudopilin gene *gspG* (dark green) under the control of an active promoter. This strain expresses *gspG* roughly 10-fold more than WT (Fig. S2) and most likely forms an overextended pseudopilus that is predicted to reduce cargo export. In both mutants, the T2SS complex is still predicted to form. (c) CFBP2957 *gspE_K274A_* cannot export polygalacturonase and is resistant to phiAP1. Ten-fold serial dilutions of each R. solanacearum strain were spotted onto minimal media plates containing polygalacturonic acid. Phage suspensions were spotted onto a lawn of each mutant. (d) The GMI1000 *gspG* OE mutant had roughly 10-fold lower secretion of polygalacturonase compared to WT and is partially resistant to phiAP1.

The phage resistance phenotype of BIM4 and BIM30 suggests that phage phiAP1 requires the T2SS for infection. However, while both BIMs lacked T2 secretion, BIM30 also had impaired twitching motility (T4P defect). To rule out a direct role of the T4P in phage phiAP1 infection, we used a validated PilA mutant of Ralstonia pseudosolanacearum strain GMI1000 (phylotype I), which is also sensitive to phage phiAP1. GMI1000 Δ*pilA* has no twitching motility because it lacks the major pilin subunit PilA. PilA is a major component of the P. aeruginosa T4P and is important for infection by some phages (31) (Fig. 3a). Both WT GMI1000 and its *ΔpilA* mutant retained phage sensitivity, indicating that neither functional twitching motility nor the T4P are required for phiAP1 infection (Fig. 3b).

10.1128/mbio.01475-22.6 FIG S2 Cloning of *gspG* under the control of the *rplM* promoter increases *gspG* expression approximately 10-fold. Semiquantitative RT-PCR was used to measure gene expression in two independent clones of GspG OE. The stably expressed *serC* transcript is used as an endogenous control. Download FIG S2, DOCX file, 0.1 MB. Copyright © 2022 Xavier et al. 2022 Xavier et al. https://creativecommons.org/licenses/by/4.0/ This content is distributed under the terms of the Creative Commons Attribution 4.0 International license.

### Phage phiAP1 requires a functional T2SS to infect R. solanacearum.

The T2SS exports proteins by using a dynamic pseudopilus to guide cargo from the periplasm to the outer membrane secretion complex from which it is released into the extracellular space. Retraction of the pseudopilus resets the system for additional rounds of secretion (23). To determine if phage phiAP1 requires a functional T2SS for infection or merely depends on the inert T2SS complex, we made two mutants in R. solanacearum strains CFBP2957 or GMI1000 designed to interfere with the secretion process while still allowing the complex to form. First, we constructed a point mutation in the ATP-binding site of GspE (gspE_K274A_), the cytoplasmic ATPase that powers addition of pseudopilin units at the base of the growing T2SS pseudopilus (23). This single amino acid change results in a GspE protein that can still form the T2SS complex but cannot bind or cleave ATP, so a functional pseudopilus cannot be assembled (Fig. 3a) (32, 33). Consistent with our hypothesis, a mutant with this single amino acid substitution not only was unable to export either polygalacturonase or pectin methylesterase, two type II secreted enzymes, but was also completely resistant to phage phiAP1 (Fig. 3c, Fig. S1).

10.1128/mbio.01475-22.5 FIG S1 Targeted mutations in the T2SS reduce the export of pectin methylesterase. Ten-fold serial dilutions of each strain were spotted on minimal media plates containing pectin. After 24 h of growth, the colonies were rinsed off and the plates were washed with HCl. BIM1 and a GspG overexpression strain produced a clearing zone at a 10-fold higher dilution than the WT. A point mutation in *gspE* led to a loss of pectin methylesterase secretion. Download FIG S1, DOCX file, 0.2 MB. Copyright © 2022 Xavier et al. 2022 Xavier et al. https://creativecommons.org/licenses/by/4.0/ This content is distributed under the terms of the Creative Commons Attribution 4.0 International license.

Second, we made a construct that overexpresses *gspG* (coding for the major T2SS pseudopilin) using a strong ribosomal promoter (34). Because the formation of the T2SS complex is dependent on correct stoichiometry (35, 36), increasing the number of available GspG subunits has been shown to increase the length of the pseudopilus, allowing it to breach the outer membrane (37, 38) and interfering with normal T2SS function (Fig. 3a). Overexpression of *gspG* with this construct was confirmed using semiquantitative RT-PCR, with expression being roughly 10-fold higher in the mutant compared to WT (Fig. S2). As expected, secretion of polygalacturonase and pectin methylesterase in this mutant decreased approximately 10-fold (Fig. 3d, Fig. S1). Remarkably, this strain was also (mostly) resistant to phage phiAP1 (Fig. 3d). Together, these results demonstrate that phage phiAP1 requires not just the assembled proteins of the T2SS but indeed a functional and energized T2SS to successfully infect R. solanacearum.

### Phage resistance imposes a fitness cost by reducing R. solanacearum virulence.

**FIG 4:**
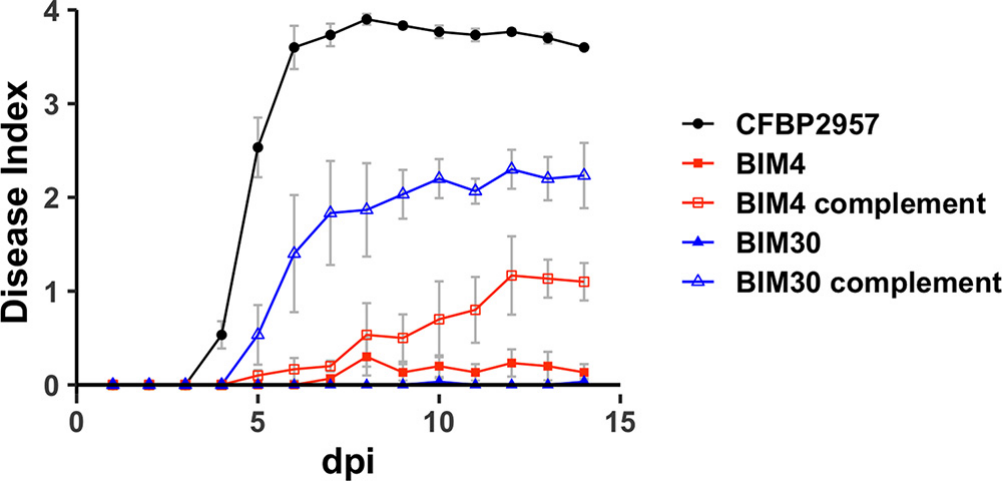
Bacteriophage-insensitive R. solanacearum mutants BIM4 and BIM30 have reduced virulence on tomato plants. Unwounded tomato plants were inoculated by pouring a 10^8^ CFU/mL suspension of R. solanacearum onto the soil. Plants were incubated in a 28°C growth chamber and rated daily on a 0 to 4 disease index scale. Each point represents the mean disease index of a total of 30 plants in 3 independent experiments. Error bars indicate standard error. R. solanacearum mutants BIM4 and BIM30 were less virulent than the phage-sensitive strains, the parent CFBP2957, and the complemented BIMs (*P* < 0.0005, repeated-measures ANOVA).

Because the T2SS translocates a wide range of plant cell wall-degrading enzymes and other proteins from the periplasm across the outer membrane, we suspected that a defective T2SS in R. solanacearum would reduce the plant pathogen’s fitness. We quantified the bacterial wilt virulence of BIM4 and BIM30 on tomato plants using a naturalistic soil soak inoculation that mimics the R. solanacearum infection process in the field. Under these conditions, WT R. solanacearum strain CFBP2957 caused significant bacterial wilt disease, killing nearly all plants within 8 days, while BIM4 and BIM30 caused little or no disease (Fig. 4). The complemented BIMs containing either the full-length *pilD* or the truncated *gspL* (658 nucleotides/219 amino acids), cloned on the low-copy-number plasmid pUFJ10, led to intermediate virulence phenotypes (Fig. 4) and were more virulent than the BIMs (BIM4 versus BIM4comp, *P = *0.024; BIM30 versus BIM30comp, *P = *0.0016, ANOVA). These data indicate that BIM4 and BIM30 are strikingly less virulent on tomato than WT R. solanacearum strain CFBP2957.

### Other BIMs also have defects in T2SS.

We analyzed an additional set of 26 BIMs isolated after exposing WT R. solanacearum strain CFBP2957 to phage phiAP1. First, we tested the twitching and T2SS phenotypes of these phage-resistant mutants. Interestingly, 20 of them (77%) also had a defect in their T2SS, suggesting that the emergence of bacteriophage-insensitive mutants with a T2SS defect is common under our laboratory conditions. Three out of the 20 T2SS-defective BIMs also lacked twitching motility. Sequencing the *pilD* genes from these three BIMs (BIM6, BIM14, and BIM19) revealed that they all had a mutation leading either to a frameshift early in the gene or to premature stop codons.

We also analyzed the virulence of these additional 26 BIMs on tomato plants using a faster infection assay in which ~200 CFU of each strain were placed on a cut leaf petiole. This introduces the bacteria directly into the plant xylem, leading to rapid, consistent disease. Bacterial wilt symptoms a week after inoculation correlated directly with the strain’s T2SS function (Table S2, Fig. S3). The 20 BIMs with a defective T2SS did not infect tomato plants, while five of the six BIMs with a functional T2SS (BIM3, BIM9, BIM17, BIM25, and BIM29) caused significant wilt disease. The phage-resistance phenotype in these five BIMs is likely due to mutations outside the T2SS system that affect other steps involved in viral infection.

10.1128/mbio.01475-22.7 FIG S3 Only T2SS-positive BIMs retain virulence. Bonny Best tomato plants (21 days old) were inoculated through a cut leaf petiole with 200 CFU of each BIM. Five plants were included per treatment. BIMs that have both functional T2SS and twitching motility are shown in red, those that are deficient in both T2SS and twitching motility are shown in grey, and BIMs that are deficient in T2SS but have WT levels of twitching motility are shown in blue. The wild-type parent strain is shown in black. Download FIG S3, DOCX file, 0.1 MB. Copyright © 2022 Xavier et al. 2022 Xavier et al. https://creativecommons.org/licenses/by/4.0/ This content is distributed under the terms of the Creative Commons Attribution 4.0 International license.

BIM1 was noteworthy because of its intermediate behavior, with secreted levels of pectin methylesterase roughly 10-fold lower than the WT strain (Fig. S1) and significantly reduced virulence compared to the fully T2SS-functional BIMs. Sequencing the genome of BIM1 revealed a 15-bp insertion in the gene coding for the T2SS inner membrane protein, GspF. This insertion added five hydrophobic amino acids (VLAAI) near the midpoint of the protein. These results bolster the connection among phage sensitivity, T2SS activity, and virulence.

## DISCUSSION

Because few effective in-field treatments for R. solanacearum exist, there has been much interest in isolating R. solanacearum-infecting phages for use as biocontrol agents (39). However, designing long-lasting biocontrol regimes likely requires a comprehensive understanding of the biology of the phages and how they interact with both R. solanacearum and their environment (40). For example, some temperate phages influence the expression of key virulence-related genes in their host. Phages of the genus *Inoviridae* have been shown to reduce the expression of virulence factors like motility and the Type III Secretion System in R. solanacearum (41 to 44), suggesting that they could be useful as a nonlethal form of disease suppression. In contrast, the inovirus φrSS1 increased R. solanacearum virulence by inducing the expression of the main quorum-sensing system regulator to allow infected cells to grow more aggressively *in planta* (45). Additionally, it is useful to understand how abiotic factors such as UV or adherence to soil particles impact phage dynamics in the field (40). Perhaps the most important factor, however, is the development of resistance to phage infection by R. solanacearum itself (22, 46). We found that R. solanacearum CFBP2957 is readily capable of developing resistance to phiAP1, echoing many previous results showing a robust ability to evolve phage resistance (46 to 49). Even when challenged by a cocktail of multiple phages, R. solanacearum can develop broad resistance to the entire cohort of phages, albeit at a fitness cost severe enough to be seen even *in vitro* (48). Carefully managing the rate of infection may help to mitigate the costs of phage resistance. The ability of a panel of lytic R. solanacearum-infecting phages to combat bacterial wilt disease both in culture and on tomato seedlings has also been examined (47). Interestingly, the phage that had the slowest replication cycle and did not completely suppress R. solanacearum growth in culture was effective at reducing bacterial wilt disease on tomato seedlings. This suggests that the phage was able to suppress R. solanacearum growth enough to promote plant health but not enough to drive resistance. Moreover, the presence of competing microbes might also impact the pressure toward resistance. When R. solanacearum was cultured with either a lytic phage, an antibiotic-producing bacterial competitor, or a combination of both, while the rate acquisition of phage resistance was unaffected by the bacterial competitor *in vitro*, a lower rate was observed in the rhizosphere, suggesting that complex biotic and abiotic factors influence R. solanacearum evolution of phage resistance (49). In our study, we only examined how R. solanacearum develops resistance to phiAP1 *in vitro*. While we found that mutations in the T2SS were the primary mechanism of resistance in our conditions, more work would be necessary to see if *R. solancearum* would use the same mechanism to develop phiAP1 resistance in the field. While the loss of the T2SS is detrimental to R. solanacearum *in planta*, it is not clear how the loss would affect its fitness in the rhizosphere in competition with other soil microbes. However, determining the major molecular pathway of resistance will aid in the further use and study of phiAP1.

**FIG 5:**
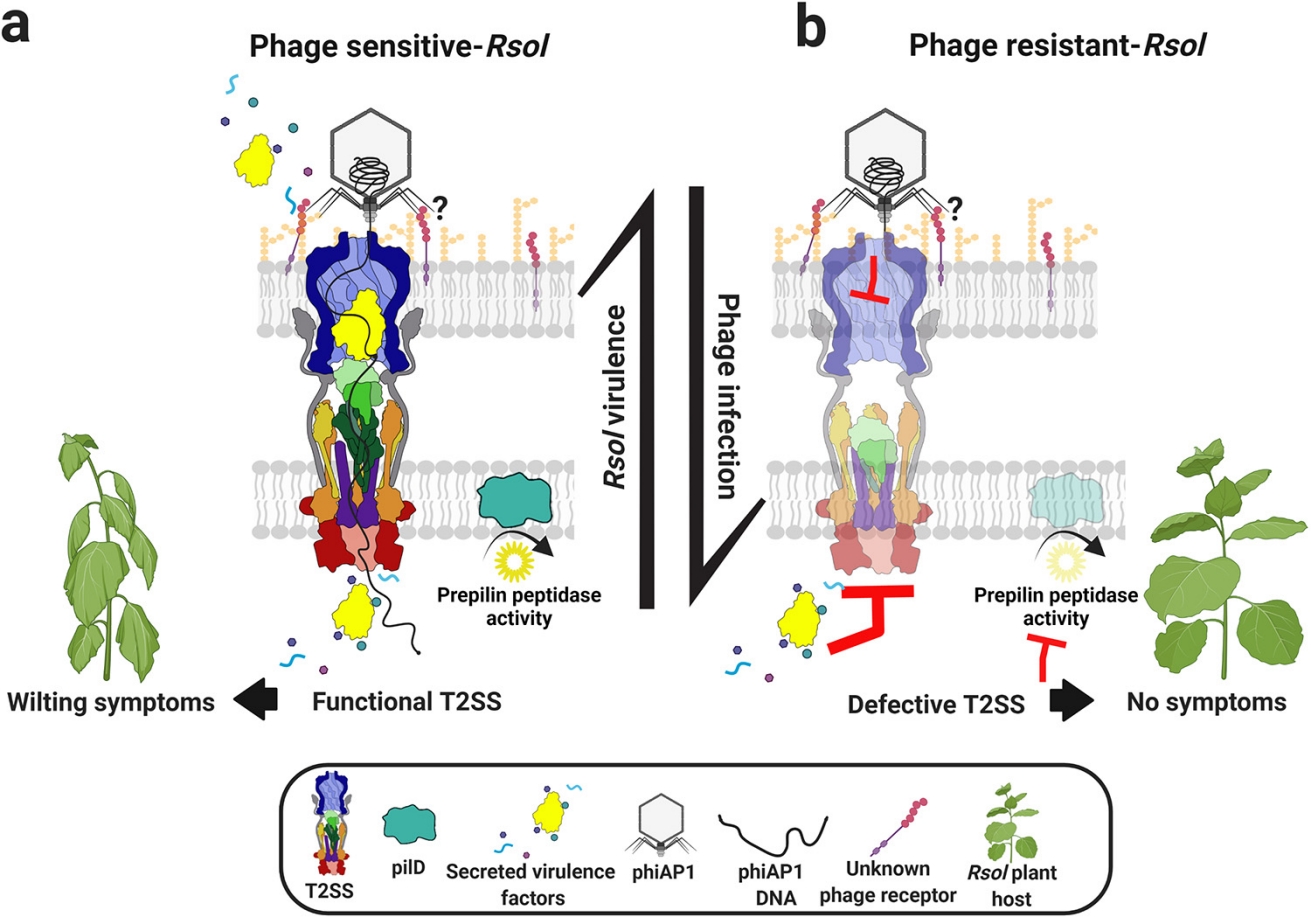
Defective T2SS-dependent phage resistance imposes a fitness cost by reducing R. solanacearum virulence. (a) After the adsorption of phage phiAP1 to its bacterial host, the phage DNA is introduced into the cell through the T2SS, which is also used for the secretion of virulence factors associated with host plant wilt symptoms. Thus, the T2SS acts as “two-way street.” (b) Once inactivated, T2SS can no longer participate in viral infection and R. solanacearum becomes phage resistant. However, the phage-resistant bacteria also lose their ability to cause plant disease as the portal is closed for the transport key macromolecules.

R. solanacearum T2SS genes and *pilD* are hot spots for phiAP1 BIM mutations, but disrupting either type II secretion or twitching motility also significantly reduces the ability of this plant pathogen to cause disease (28, 29) (Fig. 5). In P. aeruginosa, the prepilin peptidase PilD also processes prepilins for the T4P (30). The R. solanacearum PilD homolog was mutated in four BIMs, including BIM30, which is deficient in both T2SS and T4P (Table S2). This indicates that PilD also processes pseudopilins in R. solanacearum. Enzymes secreted through the T2SS help the bacterium move between adjacent xylem vessels and disrupt water flow in the host plant (50). These contributions to virulence explain why most BIMs have impaired virulence. This trade-off between phiAP1 resistance and virulence could improve the durability of phage phiAP1 as a R. solanacearum biocontrol agent. However, while the T2SS is a key component for a successful phiAP1 infection, a fraction of the analyzed BIMs had mutations unrelated to T2SS and retained their virulence in tomatoes. It has been suggested that parasite resistance evolution studies should also be performed in ecologically relevant environments to better predict the effectiveness of phage biocontrol regimes (51). Reliable biocontrol of bacterial wilt disease will likely require multiple phages, each with different host targets to reduce the incidence of resistance, which may also influence virulence.

10.1128/mbio.01475-22.2 TABLE S2 Phenotypes of several strains of Ralstonia solanacearum used in this study. Download Table S2, DOCX file, 0.02 MB. Copyright © 2022 Xavier et al. 2022 Xavier et al. https://creativecommons.org/licenses/by/4.0/ This content is distributed under the terms of the Creative Commons Attribution 4.0 International license.

Remarkably, we found that the functional activity of the T2SS was important for phiAP1 infection (Fig. 5). The fact that both BIM4 and BIM30 allow phage adsorption, supported by the requirement of a functional system, implies that the T2SS is not simply acting as a passive receptor for phiAP1 attachment. We designed T2SS mutants with reduced activity that would still allow the full assembly of the complex. First, a conserved lysine was converted to alanine in the Walker A motif of the GspE ATP binding site. An analogous substitution in EspE of Vibrio cholerae has been shown *in vitro* to have reduced ATPase activity both alone and when copurified with other T2SS inner-membrane subunits (32, 33). In R. solanacearum, this ATPase mutant could not export key T2SS enzymes and was resistant to phage phiAP1. Interfering with the stoichiometry of subunits can also alter the structure and function of the T2SS complex. Overexpressing the gene coding for the major pseudopilin GspG (or the entire *gsp* operon) results in an abnormally long pseudopilus that, unlike the native pseudopilus, crosses the outer membrane as an extracellular filament (35 to 37). We created a R. solanacearum mutant overexpressing *gspG* by using the promoter of the ribosomal protein *rplM*, which is highly expressed in culture conditions (34) like those in our T2SS and phiAP1 infection assays. To take advantage of existing genetic tools, this construct was made in the related *R. pseudosolanacearum* strain GMI1000, which is also susceptible to phiAP1 and has T2SS machinery with a high degree of homology to CFBP2957 (97% amino acid identity for GspG, 88% amino acid identify for GspL, 98% amino acid identity for GspE, 97% amino acid identity for GspD, 94% amino acid identity for pilD, 93% amino acid identity for pilA). In this mutant, *gspG* expression increased roughly 10-fold, leading to a 10-fold decrease in the secretion of T2SS enzymes and to a phage resistance phenotype. Thus, phage resistance was imparted either by preventing assembly of the native short T2SS pseudopilus or by a secretion defect via an extended hyperpseudopilus, again implicating a functional T2SS in phage infection in R. solanacearum.

BIM1 was phage resistant but kept residual T2SS activity. This mutant had a 15-bp in-frame insertion that added five hydrophobic amino acids after leucine 230 in GspF, a key component of the T2SS inner membrane platform (23). GspF is predicted to be composed of an N-terminal cytoplasmic domain, followed by a transmembrane helical hairpin, then a second cytoplasmic domain, and finally a third transmembrane helix (52). The two cytoplasmic domains interact with the cytoplasmic ATPase GspE and the cytoplasmic domain of the membrane-spanning protein GspL, respectively, to form the T2SS inner membrane platform. Alignment of the R. solanacearum GspF with its P. aeruginosa and V. cholerae homologs suggests that the 5-aa (amino acid) insertion occurred in the second TM helix (52, 53). Given that this insertion is buried in the inner membrane, it seems unlikely that this mutated region interacts directly with phage phiAP1. Instead, the mutation probably hinders phage infection through its reduced T2SS activity.

We created complements for BIM4 and BIM30, which have mutations in key elements of the T2SS, *gspL* (BIM4), and *pilD* (BIM30). Complementing BIM30 with the WT *pilD* successfully restored phage sensitivity in this BIM and largely restored its ability to cause disease in tomato plants. A truncated version of GspL (219/481 aa) was enough to reestablish sensitivity to phage phiAP1 in BIM4 but only modestly increased virulence of this mutant in tomato plants (Fig. 4). GspL is a transinner membrane protein with globular domains on cytoplasmic and periplasmic sides of the membrane (23). The substituted amino acid at position 65 is located in the cytoplasmic domain, near the site of interaction with the ATPase GspE (54). The partial restoration of function seen upon introduction of pGspL_cyto_ implies this domain may function in ATPase activation, while the residual activity of GspL_BIM4_ may serve in the periplasm. Full activity would not be expected, and is not observed, because of the interference of the defective GspL_BIM4_ in the cytoplasm and the lack of connection between the two domains via the inner membrane helix. Indeed, in the mechanistically related T4P pathway, the GspL equivalent is encoded as two separate polypeptides; PilM corresponds to the GspL_cyto_ domain while PilN encodes a single-pass transmembrane protein with a globular periplasmic domain (55).

Taken together, the analyses of dozens of phage-resistant BIMs and two site-directed mutations showed that a functional T2SS is important for phiAP1 infection. The T2SS has been shown to be a receptor for the Vibrio cholerae-infecting podovirus (56). However, the V. cholerae T2SS is likely a direct receptor for VP2, because a tail filament protein interacts with the outer membrane secretin *in vitro* and a knockout of the secretin prevented phage adsorption. Phage phiAP1 still adsorbs to both BIM4 and BIM30 (22), so it is unlikely that the static components of the R. solanacearum T2SS are only receptors for phiAP1. An energized functional T2SS is imperative for successful phiAP1 infection; it is possible that during active cycling of the T2SS, an epitope is exposed that functions as a phage receptor. However, given that it was also previously demonstrated that viral DNA replication does not occur in BIM4 (22), our data are also consistent with a molecular model in which an active T2SS is involved in phage DNA uptake. This model puts the T2SS in a category with related nanomachines like the T4P, which can serve as a natural competence pilus (57) that depends on ATPase-energized transenvelope assemblies to import DNA. While these systems generally include an active retraction ATPase, which is lacking in the T2SS, recent work has uncovered pilus retraction and DNA uptake models that are independent of this second ATPase (58, 59). Our work highlights the importance of studies on phage–host interactions to support the renaissance of phages as biocontrol agents.

## MATERIALS AND METHODS

### Bacterial isolates, phage, and growth conditions.

The wild-type phage-sensitive strain R. solanacearum CFBP2957 and its phage-resistant derivatives (BIMs) previously obtained (22) were cultured in Casamino acid-peptone-glucose (CPG) medium (60) containing casein (1 g/L), peptone (10 g/L), and glucose (5 g/L) at 28°C with shaking at 250 rpm. Bacteria were stored in CPG medium containing 15% glycerol and kept at −80°C (61). Phage phiAP1 (20) (family *Autographiviridae*, dsDNA, short tail) was propagated as described elsewhere (62), with modifications (20). The list of strains used in this study is presented in Table S3.

10.1128/mbio.01475-22.3 TABLE S3 List of strains and plasmids. Download Table S3, DOCX file, 0.02 MB. Copyright © 2022 Xavier et al. 2022 Xavier et al. https://creativecommons.org/licenses/by/4.0/ This content is distributed under the terms of the Creative Commons Attribution 4.0 International license.

### Whole-genome sequencing of R. solanacearum CFBP2957, BIM4, and BIM30.

Genomic DNA was extracted as previously described (63), except for the lysozyme step. Libraries were prepared using the Nextera XT kit, Nextera XT Index kit, and a MiSeq reagent kit v2 (Illumina) according to the manufacturer's instructions. Whole-genome sequencing (2 × 250 nucleotides) was performed on an Illumina MiSeq system. The genome of our CFBP2957 strain was assembled using Ray Assembler (64) version 2.3.1 with a k-mer size of 31 and compared to the CFBP2957 reference sequence (GenBank accession PRJEA50685). The reads from the BIMs were aligned with the CFBP2957 reference sequence and updated using the Burrows-Wheeler Alignment tool (BWA) version 0.7.10 (65). The mutations were identified using a pipeline that consisted of BAM file manipulations (merging, adding header) using Picard (version 1.123) (66) and indexed with Samtools (version 1.1) (67). Mutations were also searched using GATK (version 3.3-0) (68). The resulting .vcf files were compared manually.

### Plasmid constructs and transformation.

To verify whether the phage-sensitivity phenotype would be restored in the BIMs, the wild-type *gspL* and *pilD* genes from R. solanacearum CFBP2957 were complemented into strains BIM4 and BIM30, respectively. The replicative plasmid pUFJ10 was used in the following cloning strategy (27). An empty vector was linearized overnight at 37°C using the restriction enzyme BsrGI, which cut pUFJ10 only once and downstream of the kanamycin resistance gene (Kan^R^), and gel purified. Terminators were searched using ARNold (69) to verify whether the cloned genes could be expressed through the Kan^R^ gene promoter. Two constructs were designed for each gene. For the first construct, the *gspL* and *pilD* genes were controlled by the Kan^R^ gene promoter. For the second construct, the putative native promoter of either the *gsp* or *pil* operon was added upstream of each respective gene.

The plasmids were constructed using Gibson assembly (70), and the primers used are found in Table S4. PCR of the inserts was performed using a Q5 DNA polymerase and GC enhancer. PCR products were cleaned using the Qiagen PCR Cleanup kit and gel purified. Gibson assembly (70) was performed for 60 min using 100 ng of pUFJ10 and a 3:1 insert and vector ratio, following the manufacturer's instructions. Five μL of the Gibson assembly was transformed into E. coli NEB5α competent cells (NEB). Colony PCR was performed using a set of primers designed to target the cloned region in the plasmid (pUFJ10-3,389-F and pUFJ10-3,632-R). Resulting plasmids pGspL (*gspL* inserted in pUFJ10) and pPilD (*pilD* inserted in pUFJ10) were extracted from E. coli clones using a Qiagen Miniprep kit and confirmed by Sanger sequencing.

10.1128/mbio.01475-22.4 TABLE S4 List of primers used in this research. Download Table S4, DOCX file, 0.02 MB. Copyright © 2022 Xavier et al. 2022 Xavier et al. https://creativecommons.org/licenses/by/4.0/ This content is distributed under the terms of the Creative Commons Attribution 4.0 International license.

### Preparation of electrocompetent R. solanacearum cells.

The three strains (CFBP2957, BIM4, and BIM30) were cultivated in 200 mL of modified CPG broth (1 g/L Casamino acids, 2 g/l peptone, 0.5 g/l glucose) incubated at 30°C and shaken at 200 rpm until an OD_600nm_ of 0.8 was reached. The cultures were then transferred to a centrifuge bottle and kept on ice for 30 min. The cultures were centrifuged at 4,000 rpm for 20 min at 4°C and the supernatants discarded. The pellets were washed with 100 mL of a cold 10% glycerol solution, followed by centrifugation at 4,000 rpm for 10 min, at 4°C. This step was repeated once more, and, subsequently the pellet was carefully resuspended through successive washes using 2 mL of cold 10% glycerol solution. This process was repeated until the pellet was completely homogenized and resuspended. Several 50 μL aliquots of competent cells were transferred to 1.5-mL screw cap vials, flash frozen and stored at −80°C.

### Transformation of R. solanacearum strains.

Plasmid DNA (500 ng) was added to a 0.2-cm cuvette followed by 50 μL of electrocompetent cells. R. solanacearum CFBP2957, BIM4 (CFBP2957Δ*gspL*), and BIM30 (CFBP2957Δ*pilD*) were electroporated with either pUFJ10 empty vector, pGspLG1 (or pGspLRG), or pPilDG1 (or pPilDRG), respectively. To verify the competence of the cells, BIM4 and BIM30 were also electroporated with empty vector pUFJ10. Electroporation was performed using 200 Ω resistance, 25 μF capacitance, and a voltage of 2.5 kV (Bio-Rad). Immediately after electroporation, 950 μL of cold CPG broth was added to the cuvette. The electroporated cells were gently mixed and transferred to a microcentrifuge tube, where they were kept on ice for 10 min. The cells were then incubated for 4 h at 30°C with 200 rpm for recovery. The cell suspensions were subsequently plated on CPG agar (CPG broth with 1.5% agar), with or without 50 μg/mL of kanamycin.

### PCR screening of the complemented R. solanacearum clones.

To confirm the presence of the plasmids in R. solanacearum cells, five colonies were selected for each construction. Each colony was added to 100 μL of sterile water and heat-treated at 95°C for 5 min. Then, 3 μL of each heat-treated cell suspension was used as a template. PCRs were performed using *Taq* DNA polymerase (BioBasics) and GC enhancer at an annealing temperature at 66°C.

### Creation of mutants of *gspE* and *gspG* in R. solanacearum.

*gspE* was replaced in its native locus with an ATPase mutant using the positive selection suicide vector pUFR80 (71). The WT version of *gspE* was PCR amplified from the genomic DNA of R. solanacearum CFBP2957 using primers gspE region_F/R and inserted into the pUFR80 vector digested with XbaI and EcoRI using Gibson assembly (70). A mutated version of pUFR80-*gspE* was made using a QuikChange mutagenesis protocol gspE_K274A__F/R primers (Agilent). The pUFR80-gspE_K274A_ was then transformed into the WT R. solanacearum. Transformants were first screened on CPG supplemented with kanamycin (50 μg/mL) and counterselected on CPG supplemented with 5% wt/vol sucrose. Sucrose-resistant colonies were screened for T2SS on polygalacturonase activity plates (29) and confirmed by sequencing *gspE*.

To create a strain that overexpresses *gspG*, the *gspG* and the *rplM* promoter were PCR amplified from the genomic DNA of R. solanacearum CFBP2957 using primer pairs gspG_F/R and rplM_F/R, respectively. Resulting PCR products were cloned into the AvrII-XbaI linearized plasmid pRCK-GWY (72) using Gibson assembly. The resulting plasmid was confirmed by sequencing, transformed into *R. pseudosolanacearum* GMI1000, and selected on CPG supplemented with kanamycin (50 μg/mL). Overexpression of *gspG* was confirmed using semiquantitative RT-PCR. RNA was extracted from roughly 10^9^ cells from an overnight culture in CPG. The cells were pelleted and resuspended in 400 μL of ice-cold Tris-EDTA (TE) solution at pH 8 with 1 mg/mL lysozyme, 80 μL of 10% SDS, and 0.25 μL of SUPERase-In RNase Inhibitor (Invitrogen). The mixture was incubated at room temperature while shaking for two min. RNA was extracted from this lysate using a Zymo Quick-RNA Miniprep kit (Zymo Research). DNA was removed from the resulting materials using the DNA-Free DNA removal kit (Invitrogen). cDNA was synthesized using the Superscript VILO cDNA synthesis kit (Invitrogen, Carlsbad, CA, USA). This cDNA was serial diluted 10-fold four times and used as a template for semiquantitative RT-PCR. Reactions were conducted using GoTaq Green PCR.

### Phage-sensitivity assays.

Sensitivity to phage phiAP1 was assayed using the wild-type strains CFBP2957 and GMI1000 as well as the BIMs and their complemented derivatives. Phage sensitivity of mutants with deletions of other components of the type II secretion system (CFBP2957Δ*gspE*) or the type IV pili (GMI1000Δ*pilA*::tetA^R^) was also tested. To do so, 250 μL of an overnight bacterial culture was added to 3 mL of CPG soft agar (0.75% agar) and left to dry. Then, spot tests were performed by adding 5 μL of the phage lysate as well as of serially diluted preparations of the phage lysate on the plates that contained the inoculated bacterial culture. Plates were incubated at 30°C for 24 h. The presence of phage plaques or clear lysis zones indicated phage sensitivity. Double layer plaque assays were performed by pouring CPG soft agar containing 250 μL of the overnight bacterial culture with 100 μL of phage dilutions on CPG agar plates.

### Twitching motility test.

Twitching motility assays were conducted as previously described (28). Briefly, a 10-fold serial dilution of bacterial suspensions in water were spotted onto CPG plates with 0.08% agar. Colonies were allowed to grow for 16 h, and the colony margins were imaged under a light microscope.

### Tomato disease assays.

The virulence of BIM mutants was measured on wilt-susceptible tomato plants (cv. Bonny Best), as previously described (73). For soil soak inoculations, overnight cultures of each R. solanacearum strain were resuspended in water to a final concentration of 10^8^ CFU/mL. Fifty mL of this suspension was poured directly onto the soil of each pot containing one unwounded 17-day-old plant in a 28°C growth chamber with a 12-h photoperiod. Disease was rated daily for 2 weeks on a 0 to 4 Disease Index scale where 0 = no leaflets wilted, 1 = 1 to 25% wilted, 2 = 26 to 50% wilted, 3 = 51 to 75% wilted, and 4 = 76 to 100% wilted (74). Soil soak assays were replicated in three independent experiments containing 10 plants per strain (total *n* = 30 plants per strain). For petiole inoculations, roughly 200 CFU of each strain suspended in water was introduced to 21-day-old plants through a cut petiole. Disease was rated daily for 1 week using the rating scheme described above. Five plants were inoculated per strain.

### Type II Secretion System assays.

Activity of the T2SS was tested as described (29). Bacterial cultures were suspended in water to a concentration of 10^5^ CFU/mL and spotted on modified BMM plates (0.5 g/l ammonium sulfate, 3.4 g/l KH_2_PO_4_, 0.125 g/l FeSO_4_.7H_2_O, 250 μM MgSO_4_) with 0.1% yeast extract, 0.5% glycerol, and 1.6% agar. Pgl media (29) was prepared by adding modified BMM and 0.5% polygalacturonic acid (final concentration), while modified BMM, 0.1 mM CaCl_2_ 1 mM MgSO_4_, and 0.5% pectin were added to Pme media (29). After 24 h growth, the plates were flooded with 2N HCl.

### Whole-genome sequencing of T2SS positive mutant.

Genomic DNA was extracted from BIM1 using the Epicentre MasterPure gDNA extraction kit (Epicentre). Whole-genome sequencing was conducted at the Microbial Genome Sequencing Center (MIGS, Pittsburgh, PA, USA) using an Illumina NextSeq 550 platform. Reads were cleaned using Trimmomatic version 0.39 with a sliding window of four and trimming bases with a Phred score below 20. Trimmed reads were then assembled on the CFBP2957 genome using BWA version 0.7.17. The assemblies were then indexed using Samtools version 1.9. Variants were detected, and a .vcf file was produced using bcftools version 1.9. Variants were compared manually, as described above.
