# Supplementary material for: In through the Out Door: A Functional Virulence Factor Secretion System Is Necessary for Phage Infection in Ralstonia solanacearum
Source: mBio. 2022 Oct 31;13(6):e01475-22. doi: 10.1128/mbio.01475-22 (PMC9765573; doi:10.1128/mbio.01475-22)
Supplement: TABLE S1 [file mbio.01475-22-s0004.docx]

**TABLE S1** Mutation in the BIMs of *Ralstonia solanacearum* strain CFBP2957.

* Gene in the antiparallel strand.

Locus ID are cited and presented in *Ralstonia* genomes Database at http://iant.toulouse.inra.fr/R.solanacearumti or http://sequence.toulouse.inra.fr/R.solanacearum.
