# Supplementary material for: In through the Out Door: A Functional Virulence Factor Secretion System Is Necessary for Phage Infection in Ralstonia solanacearum
Source: mBio. 2022 Oct 31;13(6):e01475-22. doi: 10.1128/mbio.01475-22 (PMC9765573; doi:10.1128/mbio.01475-22)
Supplement: FIG S2 [file mbio.01475-22-s0002.docx]

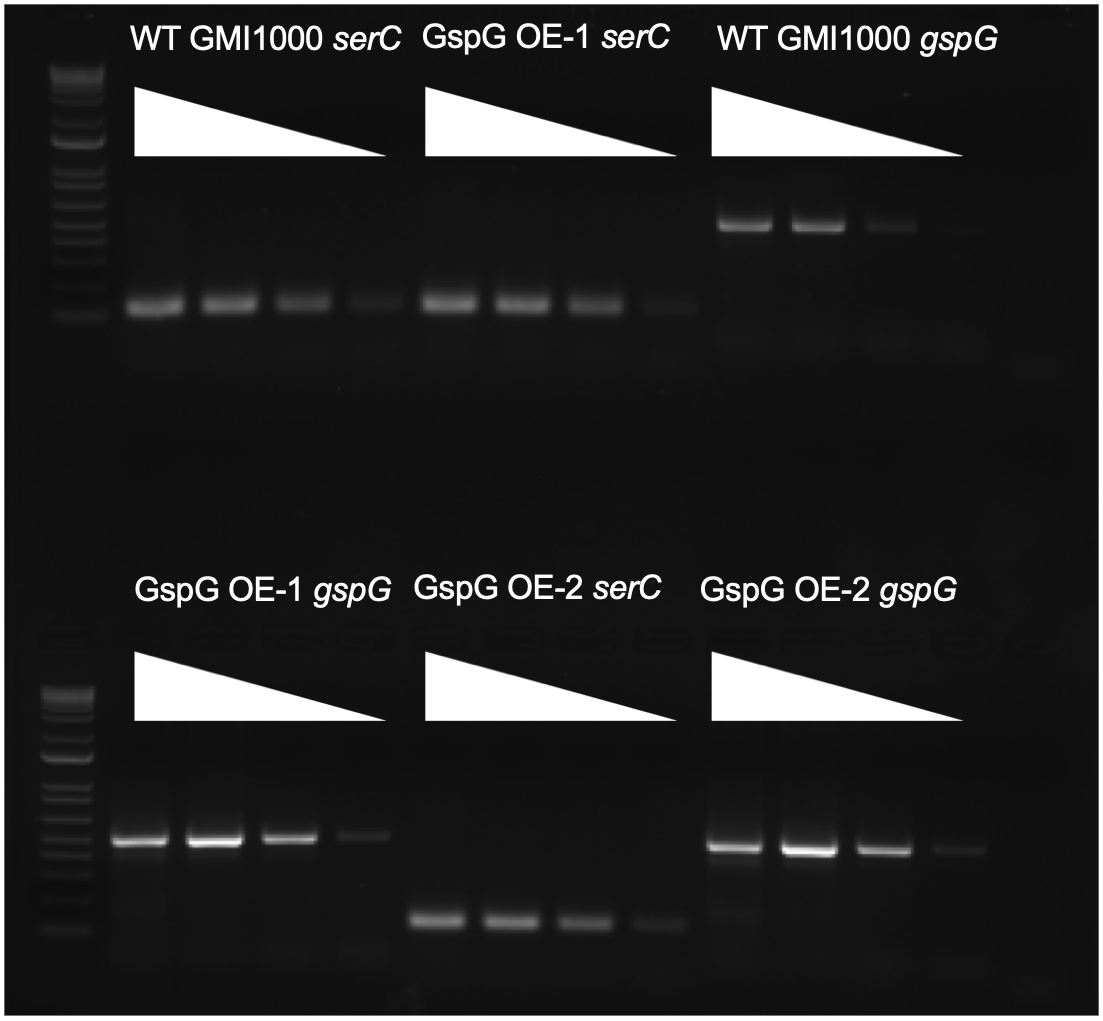


**FIG S2.** Cloning of *gspG* under the control of the *rplM* promoter increases *gspG* expression approximately ten-fold. Semi-quantitative RT-PCR was used to measure gene expression in two independent clones of GspG OE. The stably expressed *serC* transcript is used as an endogenous control.
