# Supplementary material for: In through the Out Door: A Functional Virulence Factor Secretion System Is Necessary for Phage Infection in Ralstonia solanacearum
Source: mBio. 2022 Oct 31;13(6):e01475-22. doi: 10.1128/mbio.01475-22 (PMC9765573; doi:10.1128/mbio.01475-22)
Supplement: FIG S1 [file mbio.01475-22-s0001.docx]

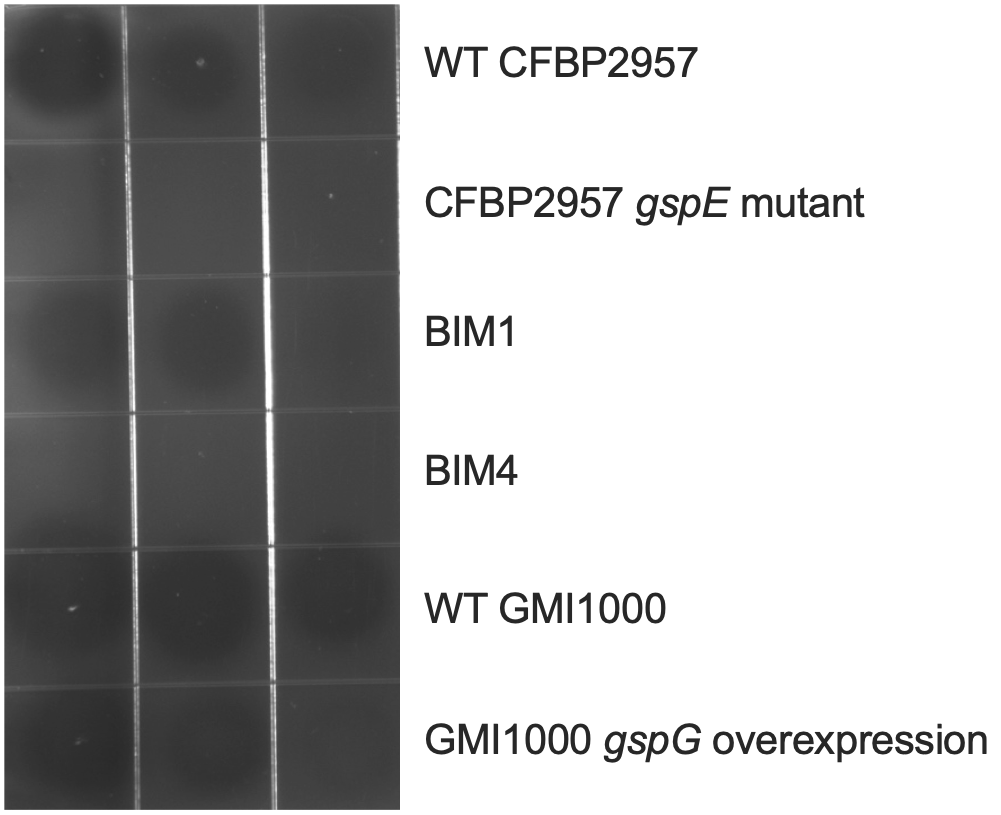


**FIG S1** Targeted mutations in the T2SS reduce the export of pectin methylesterase. Ten-fold serial dilutions of each strain were spotted on minimal media plates containing pectin. After 24 hours of growth, the colonies were rinsed off and the plates were washed with HCl. BIM1 and a GspG overexpression strain produced a clearing zone at a 10-fold higher dilution than the WT. A point mutation in *gspE* led to a loss of pectin methylesterase secretion.
