# Supplementary material for: In through the Out Door: A Functional Virulence Factor Secretion System Is Necessary for Phage Infection in Ralstonia solanacearum
Source: mBio. 2022 Oct 31;13(6):e01475-22. doi: 10.1128/mbio.01475-22 (PMC9765573; doi:10.1128/mbio.01475-22)
Supplement: FIG S3 [file mbio.01475-22-s0003.docx]

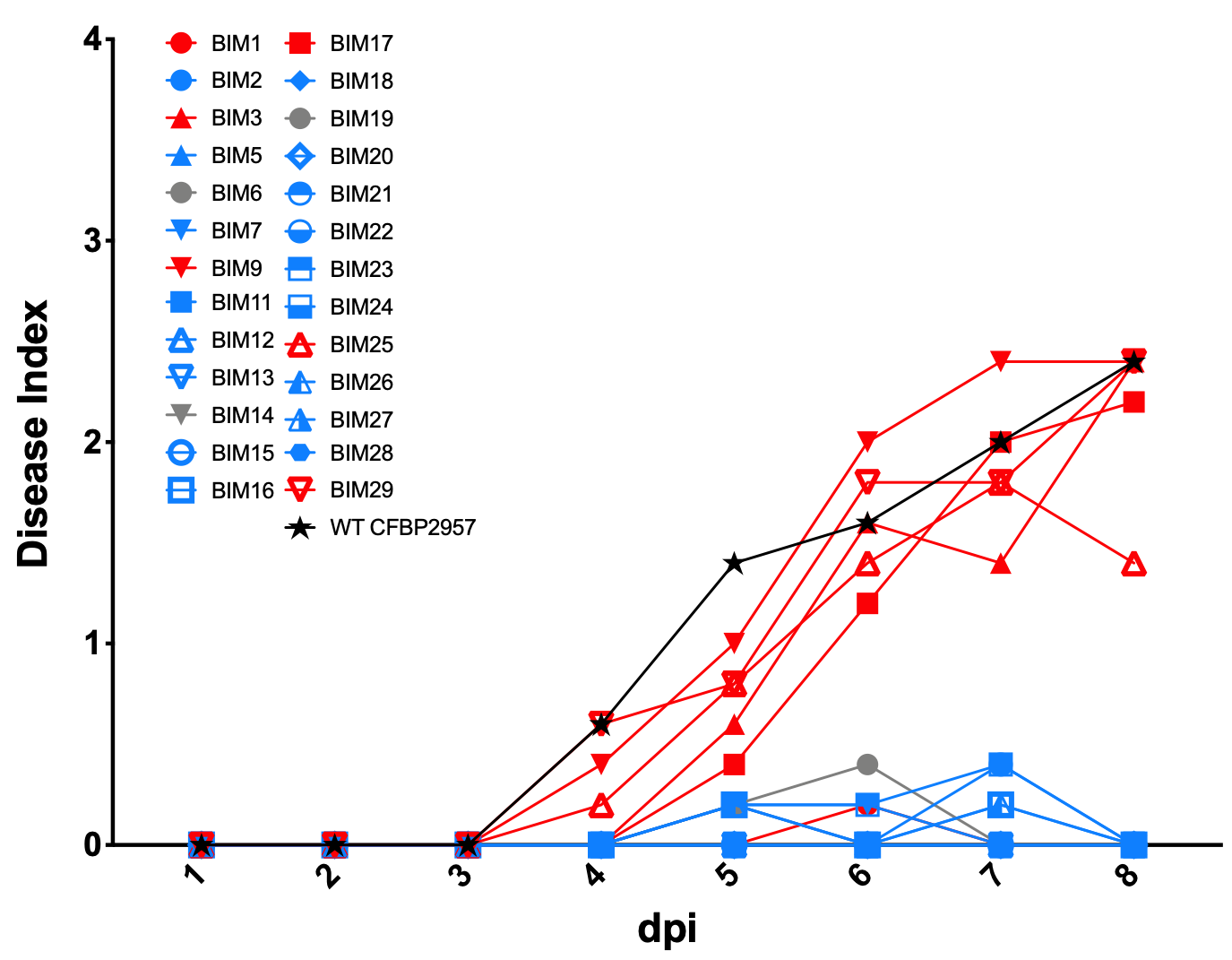


**FIG S3** Only T2SS-positive BIMs retain virulence. Bonny Best tomato plants (21-day-old) were inoculated through a cut leaf petiole with 200 CFU of each BIM. Five plants were included per treatment. BIMs that have both functional T2SS and twitching motility are shown in red, those that are deficient in both T2SS and twitching motility are shown in grey, and BIMs that are deficient in T2SS but have WT levels of twitching motility are shown in blue. The wild-type parent strain is shown in black.
