# Supplementary material for: In through the Out Door: A Functional Virulence Factor Secretion System Is Necessary for Phage Infection in Ralstonia solanacearum
Source: mBio. 2022 Oct 31;13(6):e01475-22. doi: 10.1128/mbio.01475-22 (PMC9765573; doi:10.1128/mbio.01475-22)
Supplement: TABLE S2 [file mbio.01475-22-s0005.docx]

**TABLE S2** Phenotypes of several strains of *Ralstonia solanacearum* used in this study.

| **Strain** | **phiAP1 infection** | **Twitching motility^(a)^** | **Type II Secretion^(b)^** | **Virulence ^(c)^** | **Mutation site** |
| --- | --- | --- | --- | --- | --- |
| **WT CFBP2957** | **+** | **+** | **+** | **+** | N/A; CFBP2957 (phylotype II sequevar 36) was isolated from a wilting tomato plant in Martinique, French West Indies |
| **WT GMI1000** | **+** | **+** | **+** | **+** | N/A; GMI1000 (phylotype I sequevar 18) was isolated from a wilting tomato plant in French Guyana |
| **∆pilA (GMI1000)** | **+** | **-** | **+** | **-** | *pilA* (RSc0558) (Pilin) |
| **GspG OE (GMI1000)** | **-** | Untested | **+/- ^(d)^** | Untested | CFBP2957 *gspG* expressed by *rplM* promoter at the *Rs* GMI1000 *att* site (31) |
| **GspE_K274A_** | **-** | Untested | **-** | **-** | *gspE* (RCFBP_10322) (T2SS ATPase) |
| **BIM1** | **-** | **+** | **+/- ^(d)^** | **-** | GspF (RCFBP_10320) (T2SS inner membrane protein) |
| **BIM2** | **-** | **+** | **-** | **-** | Unknown |
| **BIM3** | **-** | **+** | **+** | **+** | Unknown |
| **BIM4** | **-** | **+** | **-** | **-** | GspL (RCFBP_10326) (T2SS structural) |
| **BIM5** | **-** | **+** | **-** | **-** | Unknown |
| **BIM6** | **-** | **-** | **-** | **-** | *pilD* (RCFBP_10633) (prepilin peptidase)^(e)^ |
| **BIM7** | **-** | **+** | **-** | **-** | Unknown |
| **BIM9** | **-** | **+** | **+** | **+** | Unknown |
| **BIM11** | **-** | **+** | **-** | **-** | Unknown |
| **BIM12** | **-** | **+** | **-** | **-** | Unknown |
| **BIM13** | **-** | **+** | **-** | **-** | Unknown |
| **BIM14** | **-** | **-** | **-** | **-** | pilD (RCFBP_10633) (prepilin peptidase)^(e)^ |
| **BIM15** | **-** | **+** | **-** | **-** | Unknown |
| **BIM16** | **-** | **+** | **-** | **-** | Unknown |
| **BIM17** | **-** | **+** | **+** | **+** | Unknown |
| **BIM18** | **-** | **+** | **-** | **-** | Unknown |
| **BIM19** | **-** | **-** | **-** | **-** | pilD (RCFBP_10633) (prepilin peptidase)^(e)^ |
| **BIM20** | **-** | **+** | **-** | **-** | Unknown |
| **BIM21** | **-** | **+** | **-** | **-** | Unknown |
| **BIM22** | **-** | **+** | **-** | **-** | Unknown |
| **BIM23** | **-** | **+** | **-** | **-** | Unknown |
| **BIM24** | **-** | **+** | **-** | **-** | Unknown |
| **BIM25** | **-** | **+** | **+** | **+** | Unverified |
| **BIM26** | **-** | **+** | **-** | **-** | Unknown |
| **BIM27** | **-** | **+** | **-** | **-** | Unknown |
| **BIM28** | **-** | **+** | **-** | **-** | Unknown |
| **BIM29** | **-** | **+** | **+** | **+** | Unknown |
| **BIM30** | **-** | **-** | **-** | **-** | pilD (RCFBP_10633) (prepilin peptidase) |

a. Twitching motility was assessed by observing the colony margins of *R. solanacearum* colonies grown on low-percentage agar plates.

b. T2SS was assessed by growing each strain on minimal media plates containing polygalacturonic acid, a polymer that is degraded by a T2SS exported enzyme. T2SS positive colonies produce a zone of clearing when plates are flooded with HCl.

c. Virulence on tomato of *R. solanacearum* BIM strains was assayed by inoculating ~200 CFU through a cut leaf petiole and visually rating bacterial wilt disease for 7 days. Five plants were inoculated per strain.

d. *R. solanacearum* BIM1 and GspG overexpressing strains had a ten-fold reduced T2SS activity fold (Fig. 3C, Supplementary Fig. 1).

e. Only the *pilD* gene was sequenced for BIM6, BIM14, and BIM19, all other mutations were identified by whole genome sequencing.
