# Supplementary material for: In through the Out Door: A Functional Virulence Factor Secretion System Is Necessary for Phage Infection in Ralstonia solanacearum
Source: mBio. 2022 Oct 31;13(6):e01475-22. doi: 10.1128/mbio.01475-22 (PMC9765573; doi:10.1128/mbio.01475-22)
Supplement: TABLE S3 [file mbio.01475-22-s0006.docx]

**TABLE S3** List of strains and plasmids.

| **Plasmid** | **Description** | **References** |
| --- | --- | --- |
| pUFJ10 | GM^R^, Kan^R^ | 1 |
| pGspLG1 | pUFJ10 carrying CFBP2957 *gspL* under the control of the *Kan^R^* promoter | This study |
| pGspLRG1 | pUFJ10 carrying CFBP2957 *gspL* under the control of the native *gsp* promoter | This study |
| pPilDG1 | pUFJ10 carrying CFBP2957 *pilD* under the control of the *Kan^R^* promoter | This study |
| pPilDRG1 | pUFJ10 carrying CFBP2957 *pilD* under the control of the native *pil* promoter | This study |
| pUFR80 | Suc^S^, Kan^R^, positive selection suicide vector | 2 |
| pUFR80-gspE | pUFR80 carrying WT *gspE* | This study |
| pUFR80-gspE_K274A_ | pUFR80 carrying *gspE* with k274 replaced with alanine | This study |
| pRCK-GWY | Kan^R^, contains recombination regions matching a neutral region in the GMI1000 chromosome | 3 |
| pRCK-gspG overexpression | pRCK-GWY carrying CFBP2957 *gspG* under the control of the *rplM* promoter | This study |
| **Strain** | **Description** | **Reference** |
| *R. solanacearum* CFBP2957 | WT, Phylotype IIA, isolated from tomato in Martinique | 4 |
| *R. pseudosolanacearum* GMI1000 | WT, Phylotype I, isolated from tomato in French Guyana | 4 |
| BIM4 | CFBP2957 mutant resistant to phiAP1, in-frame mutation in *gspL* | 5 |
| BIM30 | CFBP2957 mutant resistant to phiAP1, frameshift in *pilD* | 5 |
| BIM4 comp | BIM4 complemented with pGspLG1, Kan^R^ | This study |
| BIM4+pUFJ10 | BIM4 carrying pUFJ10, Kan^R^ | This study |
| BIM30 comp | BIM30 complemented with pPilDG1, Kan^R^ | This study |
| BIM30+pUFJ10 | BIM30 carrying pUFJ10, Kan^R^ | This study |
| CFBP2957 *gspE* mut | CFBP2957 with *gspE K274A* | This study |
| *∆pilA* | GMI1000 lacking *pilA*, Tet^R^ | This study |
| GMI1000 *gspG* overexpression | GMI1000 expressing CFBP2957 *gspG* under the control of the *rplM* promoter, Kan^R^ | This study |
| *E. coli* DH5α | F– Φ80*lac*ZΔM15 Δ(*lac*ZYA-argF) U169 *rec*A1 *end*A1 *hsd*R17 (rK–, mK+) *pho*A *sup*E44 λ– *thi*-1 *gyr*A96 *rel*A1 | Invitrogen |
| *E. coli* NEB5α | Competent *E. coli* DH5α derivative | NEB |

**REFERENCES**

1. Harvey, H. *et al.* 2018. *Pseudomonas aeruginosa* defends against phages through type IV pilus glycosylation. Nat Microbiol 3:47–52.
2. Monteiro, F., Solé, M., van Dijk, I. & Valls, M. 2012. A chromosomal insertion toolbox for promoter probing, mutant complementation, and pathogenicity studies in *Ralstonia solanacearum*. Mol Plant Microbe Interact 25:557–68.
3. Durand, É. *et al.* 2005. XcpX controls biogenesis of the *Pseudomonas aeruginosa* XcpT-containing pseudopilus. J Biol Chem 280:31378–31389.

# Remenant, B., et al. 2010. Genomes of three tomato pathogens within the *Ralstonia solanacearum* species complex reveal significant evolutionary divergence. BMC Genomics 11:379.

1. Liu, H., Kang, Y., Genin, S., Schell, M. A. & Denny, T. P. 2001. Twitching motility of *Ralstonia solanacearum* requires a type IV pilus system. Microbiology 147:3215–3229.
