# Supplementary material for: In through the Out Door: A Functional Virulence Factor Secretion System Is Necessary for Phage Infection in Ralstonia solanacearum
Source: mBio. 2022 Oct 31;13(6):e01475-22. doi: 10.1128/mbio.01475-22 (PMC9765573; doi:10.1128/mbio.01475-22)
Supplement: TABLE S4 [file mbio.01475-22-s0007.docx]

**TABLE S4** List of primers used in this research.

| **Primer** | **Sequence (5’-3’)** | **Reference** |
| --- | --- | --- |
| pilD1_F | CAACGTAGCTCTTGCCGCTG | This study |
| pilD1_R | CGATCATCATCGTGATCCTG | This study |
| gspL1-F | CGTTGGAGGAAGTTGCCATAC | This study |
| gspL3-R | GCCTTCGCCGTATTGTCAT | This study |
| gspL2-F | GCTCCCAGAACAGCAGATAGA | This study |
| gspL1-R | CTCTTGGTAGCGTTTTGAC | This study |
| pilD_Gib_pUFJ-F | gttttcatggcttgttatgactgtttttttCGATCATCATCGTGATCCTGGGCGTG | This study |
| pilD_Gib_pUFJ-R | cttgctgcttggatgcccgaggcatagactGCCGTTCCTTACCGCGCCAGC | This study |
| gspL_Gib_pUFJ-F | gttttcatggcttgttatgactgtttttttGCCTTCGCCGTATTGTCATC | This study |
| gspL_Gib_pUFJ-R | cttgctgcttggatgcccgaggcatagactCGATCGTTATTGCGCGATGG | This study |
| pUFJ10_3389-F | GATGAAGGCACGAACCCAGT | This study |
| pUFJ10_3632-R | CGTAACATCGTTGCTGCTCC | This study |
| gspEregion_F | tgcatgcctgcaggtcgactCCTGGGCGATATTCCCATC | This study |
| gspEregion_F | cagctatgaccatgattacgCGCATTGATGAAGAGCACGG | This study |
| gspE_K274A__F | GACCGGGTCGGGCGCGACCACCACGCTG | This study |
| gspE_K274A__R | CAGCGTGGTGGTCGCGCCCGACCCGGTC | This study |
| rplM_F | tgcgcgagcaggggaattgcATTCTTTTCCTTGTGTCAAG | This study |
| rplM_R | cttgcatcatGATTTTTCCAAATTTGAGTCAG | This study |
| gspG_F | tggaaaaatcATGATGCAAGGCCAACTTC | This study |
| gspG_R | cgaccctagtctaagatcttTCAATTGTCCCAGTTGCC | This study |
| serC_F | CGCGCAAATACGGTGAAGTG | Hendrich et al. |
| serC_R | GTGCACAGATGCACGTAAGC | Hendrich et al. |

**REFERENCE**

1. Hendrich, C. G., Truchon, A. N., Dalsing, B. L. & Allen, C. Nitric oxide regulates the *Ralstonia solanacearum* type 3 secretion system. BioRxiv preprint doi: https://doi.org/10.1101/2020.10.26.355339.
